# Rate of misdiagnosis and clinical usefulness of the correct diagnosis in exudative neovascular maculopathy secondary to AMD versus pachychoroid disease

**DOI:** 10.1038/s41598-020-77566-1

**Published:** 2020-11-23

**Authors:** Enrico Borrelli, Marco Battista, Francesco Gelormini, Riccardo Sacconi, Lea Querques, Giovanna Vella, Chiara Viganò, Francesco Bandello, Giuseppe Querques

**Affiliations:** 1 Department of Ophthalmology, University Vita-Salute San Raffaele, Via Olgettina 60, Milan, Italy; 2 IRCCS San Raffaele Scientific Institute, Milan, Italy; 3 Ophthalmology, Department of Surgical, Medical, Molecular Pathology and of Critical Area, University of Pisa, Pisa, Italy

**Keywords:** Macular degeneration, Retinal diseases

## Abstract

The aim of this study was to explore the relative prevalence and clinical differences between age-related macular degeneration (AMD) and pachychoroid disease in patients older than 50 years with newly diagnosed exudative neovascular maculopathy, and also assess the rate of misdiagnosis between these two disorders. In this retrospective observational study, we reviewed data from patients 50 years of age and older with newly diagnosed treatment-naïve exudative macular neovascularization (MNV) secondary to AMD or pachychoroid disease. Of the 139 patients (139 eyes) who fulfilled the inclusion criteria, 35 patients were graded as being affected by pachychoroid disease complicated by exudative MNV and 104 subjects had neovascular AMD. Therefore, prevalence of pachychoroid disease complicated by exudative MNV was 25.2% (confidence interval—CI 18.2–33.2%). Mean ± SD age was 67.0 ± 8.8 years in the pachychoroid disease group and 80.6 ± 6.6 years in the neovascular AMD group (*P* < 0.0001). At baseline, BCVA was better in patients with pachychoroid disease complicated by exudative MNV (0.4 ± 0.3 LogMAR vs. 0.7 ± 0.5 LogMAR, *P* = 0.003). At the 1-year follow-up visit, BCVA was still better in patients with pachychoroid-associated MNV (0.34 ± 0.32 LogMAR vs. 0.59 ± 0.52 LogMAR; *P* = 0.005). In our study cohort, 19 patients were graded to be affected by pachychoroid disease complicated by exudative MNV even though a diagnosis of neovascular AMD was erroneously reported in their medical records at baseline. In conclusion, pachychoroid disease is a frequent cause of exudative MNV in aged patients with a high rate of misdiagnosis. A correct diagnosis may be important as these two disorders differ in terms of clinical characteristics and prognosis.

## Introduction

Age-related macular degeneration (AMD) and pachychoroid disease represent two distinct disorders sharing a conclusive common clinical pathway that emerges from a number of insults eventually leading to choroidal disturbance^1–5^. Both these disorders may be complicated by the development of an exudative macular neovascularization (MNV), an entity referred to as “exudative maculopathy”^6^. The latter feature suggests that an ischemia of the outer retina related to choroidal dysfunction may help drive the neovascular process in both AMD and pachychoroid disease^1–5,7^.

The pachychoroid disease spectrum incorporates different entities that include central serous chorioretinopathy (CSC)^8,9^, pachychoroid pigment epitheliopathy^10^, focal choroidal excavation^11^, peripapillary pachychoroid syndrome^12^, pachychoroid neovasculopathy^13,14^, and pachychoroid aneurysmal type 1 macular neovascularization^13^. More importantly, as stated above, pachychoroid disorders may be complicated by the development and exudation of secondary MNV. First, the occurrence of type 1 (sub-retinal pigment epithelium [RPE]) MNV has been reported in eyes with chronic CSC^15^. In details, Tang et al.^15^ described clinical characteristics and demographics of 9 patients with CSC and type 1 MNV. The mean age of these patients was 61 years (range 48–76 years) and the mean interval between diagnosis of CSC and detection of MNV was 139 months, these findings suggesting that MNV are more likely to develop in older patients with a longer history of CSC. Second, the occurrence of type 1 MNV has also been reported in eyes with other pachychoroid disease entities^16–18^. Pang and Freund^16^ reported cases of type 1 MNV developing in eyes with pachychoroid pigment epitheliopathy and termed this condition as “pachychoroid neovasculopathy”. In details, the authors described 3 eyes of 3 patients aged between 55 and 63 years and without previous history of CSC. Finally, eyes with pachychoroid disease may be complicated by the development of aneurysmal (polypoidal) vascular structures^17,19^.

Exudative neovascular AMD represents the leading cause of permanent damage of central vision among subjects older than 50 years. In AMD, exudation may develop from pathologic type 1, type 2 (sub-retina), or type 3 (intra-retinal) MNV^20^. However, as stated above, individuals older than 50 years may also experience exudative MNV in the setting of pachychoroid disease. While exudative neovascular AMD has been fully characterized in terms of clinical characteristics and prevalence, the incidence of exudative MNV in pachychoroid disease has not been well established.

Moreover, differentiating pachychoroid disease complicated by exudative MNV from AMD can be challenging, as these two conditions may have similar characteristics on dye angiographies (fluorescein angiography and indocyanine green angiography, FA and ICGA, respectively) and both disorders usually affect patients older than 50 years. However, improvements in choroidal imaging and advances in our understanding of the pachychoroid disease spectrum have enhanced our capability to differentiate cases with MNV-associated pachychoroid disease from neovascular AMD.

In this retrospective study we investigated patients older than 50 years with newly diagnosed treatment-naïve exudative MNV. The aim of this study was thus to explore the relative prevalence of neovascular AMD and exudative MNV secondary to pachychoroid disease in these patients. Assuming that the differential diagnosis between these two entities may be challenging, we also assessed the rate of misdiagnosis in this study cohort. Finally, we investigated differences in terms of demographics, clinical characteristics, and prognosis between these two disorders.

## Methods

This study was a retrospective cross-sectional analysis. The San Raffaele Ethics Committee approved this retrospective cohort study. The study adhered to the 1964 Helsinki declaration and its later amendments. Informed consent was obtained from all subjects and it was authorized by the San Raffaele Ethics Committee.

Consecutive subjects 50 years of age and older with newly diagnosed treatment-naïve exudative MNV secondary to AMD or pachychoroid disease in the period between January 2018 and December 2019 were identified from the medical records of the “Medical Retina and Imaging Unit” at San Raffaele Scientific Institute. Clinical diagnosis was determined by clinical examination and multimodal imaging assessment^7^. At the time of diagnosis, all patients underwent a complete ophthalmologic examination including best-corrected visual acuity (BCVA) assessment, slit lamp biomicroscopy and fundus examination by an experienced retina specialist, structural optical coherence tomography (OCT) scans and fluorescein and indocyanine green angiographies. Noteworthy, the starting diagnosis (referral diagnosis in cases of patients firstly diagnosed in other retina units and/or general ophthalmologists) was also recorder for each patient.

Exclusion criteria included: (1) MNV secondary to high myopia, trauma, angioid streaks, uveitis, or any other disorder not including AMD and pachychoroid disease; (2) history of previous retinal surgeries and anti-vascular endothelial growth factor (VEGF) injections; and (3) history or evidence of other retinal and optic nerve disorders.

Structural OCT and dye angiography imaging was performed with the Heidelberg Spectralis HRA + OCT device (Heidelberg Engineering, Heidelberg, Germany). The OCT imaging session included 6 radial linear SD-OCT scans centered on the fovea, each composed by 25 averaged OCT B-scans (768 A-scans per line) at 30°. A minimum signal strength of 25 was required to the OCT images to be included, as recommended by the manufacturer^21^.

### Grading process

Structural OCT and dye angiography (FA and ICGA) images were reviewed by two independent and experienced readers (EB and MB). The grading process was aimed at distinguishing cases with MNV-associated pachychoroid disease from neovascular AMD. Although there is no established consensus on the diagnosis of pachychoroid disease complicated by exudative MNV, the two graders employed the criteria previously described for neovascular exudative maculopathy phenotyping^7,22,23^, that were slightly modified (see below). In details, a diagnosis of pachychoroid disease was made if all the following criteria were confirmed: (1) subfoveal choroidal thickness ≥ 200 μm in both eyes; (2) no evidence of drusen—i.e. the presence of pachydrusen, that were fully described by Spaide^24^, was instead allowed, in contrast with previous papers^7,22,23^; and (3) history of CSC or presence of one of the imaging characteristics among choroidal vascular hyperpermeability, RPE changes consistent with pachychoroid disease, and dilated choroidal vessels or choroidal thickening at the level of MNV.

Graders performed grading in an independent and blinded fashion. Thereafter they met to compare level of agreement, and disagreements were resolved by further discussion and open adjudication to yield a single assessment for each case. In those cases in which the two graders did not agree on a single consensus result, the final decision was made by a third expert in retinal disorders (GQ).

Finally, a single grader (MB) evaluated OCT images at baseline for qualitative and quantitative features, including subfoveal choroidal thickness and type of MNV.

### Statistical analysis

To detect departures from normality distribution, a Shapiro–Wilk’s test was performed for all variables. The analysis included descriptive statistics (using Microsoft Office Excel software; version 14.0, 2010, Redmond, WA, USA) for demographics and main clinical data. Means and standard deviation (SD) were computed for all quantitative variables. The Student’s t-test for independent samples was used to compare age between neovascular AMD and pachychoroid disease groups. Other quantitative variables were compared with one-way analysis of covariance (ANCOVA), by introducing age as covariate. Qualitative variables were compared using Fisher’s exact test.

Statistical calculations were performed using Statistical Package for Social Sciences (version 20.0, SPSS Inc., Chicago, IL, USA). The chosen level of statistical significance was *P* < 0.05.

## Results

Table 1 summarizes demographics and clinical characteristics of enrolled patients. All patients were Caucasian. Of the 139 patients (139 eyes) with newly diagnosed treatment naïve exudative MNV who fulfilled inclusion criteria, 35 patients (29 males) were graded as being affected by MNV-associated pachychoroid disease and 104 subjects (40 males) were affected by neovascular AMD. The two graders agreed in their observations in 133 out of 139 eyes. The agreement for the remaining 6 eyes was obtained after open adjudication.

**Table 1:** Characteristics of enrolled patients.

	Neovascular AMD	Pachychoroid disease with MNV	*P* value
Number of eyes (patients)	104 (104)	35 (35)	–
Age, years	80.7 ± 6.6	67.0 ± 8.8	< 0.0001^a^
**Gender**			
M, n (%)	40 (38.5%)	29 (82.9%)	< 0.0001^b^
F, n (%)	64 (61.5%)	6 (17.1%)	
Baseline BCVA, LogMAR	0.7 ± 0.5	0.4 ± 0.3	0.003^c^
Subfoveal choroidal thickness, µm	152.5 ± 74.4	387.6 ± 81.9	< 0.0001^c^

Quantitative values are expressed in mean ± SD (standard deviation).

*BCVA* best corrected visual acuity (logMAR [logarithm of the minimum angle of resolution]).

^a^T-test.

^b^Fischer’s exact test.

^c^one-way analysis of covariance (ANCOVA) with gender and age as covariates.

Therefore, prevalence of pachychoroid disease complicated by exudative MNV in aged patients (> 50 years) in our study cohort was 25.2% (confidence interval—CI 18.2–33.2%). Mean ± SD age was 67.0 ± 8.8 years [range 50–84 years] in the pachychoroid disease group and 80.7 ± 6.6 years [range 68–100 years] in the neovascular AMD group (*P* < 0.0001).

At baseline, BCVA was 0.4 ± 0.3 LogMAR (Snellen equivalent of approximately 20/50) in eyes affected by pachychoroid disease and MNV and 0.7 ± 0.5 LogMAR (Snellen equivalent of approximately 20/100) in the eyes with neovascular AMD (*P* = 0.003).

**Figure 1:**
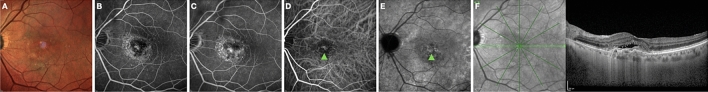
Images from a patient with a type 1 macular neovascularization secondary to age-related macular degeneration. (**A**) Multicolor image demonstrates regions of retinal pigment epithelium changes in the macular area. (**B**,**C**) Early- and late-phase fluorescein angiography (FA) images displays hyperfluorescent areas with leakage. (**D**,**E**) Early- and late-phase indocyanine green angiography (ICGA) show macular hyperfluorescence (highlighted with green head arrows) with late staining. (**F**) A fibrovascular pigment epithelial detachment associated with subretinal fluid and hyperreflective material is detected with structural OCT.

**Figure 2:**
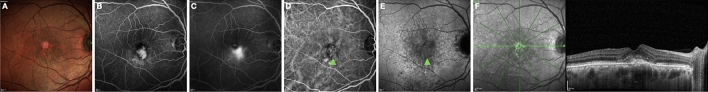
Multimodal imaging of a type 1 macular neovascularization associated with pachychoroid disease. (**A**) Retinal pigment epithelium alterations within the macula were detected using multicolor imaging modality. (**B**,**C**) Early- and late-phase fluorescein angiography (FA) demonstrates a macular hyperfluorescent area with leakage. (**D**,**E**) Early- and late-phase indocyanine green angiography (ICGA) displays the presence of an hyperfluorescent lesion (highlighted with green head arrows) with dye washout in the late phase. (**F**) A fibrovascular pigment epithelium detachment with subretinal hyperreflective material is detected with structural OCT.

Considering the neovascular AMD group, 76 out of 104 eyes displayed the presence of type 1 MNV (73.1%), while 11 (10.6%) and 17 (16.3%) eyes exhibited findings consistent with type 2 and type 3 MNV, respectively (Fig. 1). Conversely, pachychoroid eyes were all characterized by type 1 MNV (Fig. 2). Within cases with type 1 MNV and pachychoroid disease, the aneurysmal type 1 subtype (also known as polypoidal choroidal vasculopathy) was identified in 7 eyes (17.1%).

### Rate of misdiagnosis

In our study cohort, there were 104 patients (104 eyes) with neovascular AMD according to our grading in agreement with their medical record; while 19 patients (19 eyes—15.4%) were graded to be affected by pachychoroid disease complicated by exudative MNV even though a diagnosis of neovascular AMD was reported in their baseline medical records, according to the starting diagnosis. All patients with pachychoroid disease and exudative MNV in their medical record were confirmed to be affected by this disease by our grading. In a comparison analysis between patients with a correct diagnosis of pachychoroid disease complicated by exudative MNV and subjects with pachychoroid disease misclassified as neovascular AMD, the latter group was significantly older (Table 2).

**Table 2:** Characteristics of patients with pachychoroid neovasculopathy.

	Pachychoroid disease with MNV correctly diagnosed	Pachychoroid disease with MNV misclassified as neovascular AMD	*P* value
Number of eyes (patients)	16 (16)	19 (19)	–
Age, years	63.0 ± 7.5	70.3 ± 8.5	0.012^a^
**Gender**			
M, n (%)	13 (81.2%)	16 (84.2%)	0.582^b^
F, n (%)	3 (18.8%)	3 (15.8%)	
Subfoveal choroidal thickness, µm	405.3 ± 99.0	372.7 ± 63.3	0.247^a^

Quantitative values are expressed in mean ± SD (standard deviation).

^a^T-test.

^b^Fischer’s exact test.

### Longitudinal assessment of patients

An additional analysis was performed on patients with at least 1 year of follow-up after baseline. This analysis was aimed at assessing clinical characteristics and functional results at 1 year of follow-up and to compare them between the two groups. This analysis included 23 patients with pachychoroid disease complicated by exudative MNV and 79 patients with neovascular AMD.

Mean ± SD anti-VEGF injections during the first year of follow-up after baseline was 4.1 ± 2.1 [range 0–7 injections] in the pachychoroid disease group and 5.2 ± 2.3 [range 1–11 injections] in the neovascular AMD group (*P* = 0.128). One patient with pachychoroid disease did not undergo anti-VEGF therapy during the first year of follow-up as he refused this treatment and was treated with verteporfin photodynamic therapy (vPDT). Moreover, within the pachychoroid disease group, 7 out of 23 patients (30.4%) also underwent vPDT during the first year of follow-up.

At the 1-year follow-up visit, BCVA was still better in patients with pachychoroid disease complicated by exudative MNV (0.34 ± 0.32 LogMAR, Snellen equivalent of approximately 20/40), as compared with patients with neovascular AMD (0.59 ± 0.52 LogMAR, Snellen equivalent of approximately 20/70; *P* = 0.005). Structural OCT signs of exudation—i.e. presence of subretinal and/or intraretinal fluid—resolved within the first year of follow-up in 13 out of 23 (56.5%) patients with MNV-associated pachychoroid disease and 45 out of 79 (57.0%) patients with neovascular AMD (*P* = 0.715). Mean ± SD time of resolution was 5.5 ± 2.8 months and 4.7 ± 2.2 months for pachychoroid disease and neovascular AMD patients, respectively (*P* = 0.315).

## Discussion

In this retrospective investigation, we explored consecutive patients older than 50 years with newly diagnosed treatment-naïve exudative neovascular maculopathy (neovascular AMD or pachychoroid disease complicated by exudative MNV). Overall, in our study cohort of Caucasian patients, pachychoroid disease was revealed to be a frequent cause of exudative MNV, as this entity may represent more than one quarter of all cases. Furthermore, our analysis provided evidence that pachychoroid disease complicated by exudative MNV may be often misclassified as neovascular AMD. Our findings highlight the significance of an appropriate differential diagnosis between these two entities as this may significantly influence disease management and visual prognosis.

Progresses in retinal imaging techniques have furnished important tools to differentiate pachychoroid disease complicated by exudative MNV from neovascular AMD. In a multimodal analysis using both structural OCT and dye angiography, Miyake et al.^7^ evaluate 200 consecutive Asian patients diagnosed with pachychoroid disease and exudative MNV or neovascular AMD in order to estimate the relative prevalence of these two entities. In the latter study, pachychoroid disease was observed in 39 (19.5%; confidence interval [CI] 14.0–25.0%) individuals.

We add to the literature by reporting the prevalence of pachychoroid disease with exudative MNV relative to neovascular AMD in a study cohort of Caucasian subjects older than 50 years with newly diagnosed, treatment-naïve exudative neovascular maculopathy. Based on our analysis, 35 patients (25.2%; CI 18.2–33.2%) were diagnosed with pachychoroid disease and exudative MNV. Therefore, our prevalence was slightly higher than that reported by Miyake et al.^7^ in an Asian study cohort. The latter aspect may be secondary, at least in part, to differences in diagnostic criteria employed to differentiate these two entities. In details, in contrast with Miyake et al.^7^ in our study patients with drusenoid RPE elevations with characteristics resembling pachydrusen^24^ were potentially includable in the pachychoroid disease group, and not automatically graded as being affected by neovascular AMD. Furthermore, the introduction of OCT-angiography has significantly improved the diagnosis of MNV in eyes with pachychoroid disease and this aspect may have impacted on our capability to diagnose pachychoroid disease with exudative MNV. The diagnosis of MNV in patients with pachychoroid disease may be indeed challenging using ICGA, assuming that these neovascularizations are often characterized by a late washout of dye, as opposed to late staining in AMD-related MNV (Fig. 2)^25^.

Noteworthy, although it is well known that the latter disease has an higher prevalence in Asians^26^, our results suggest that also in Caucasians the prevalence of pachychoroid disease complicated by exudative MNV is much higher than that conventionally believed. In addition, at baseline, the two groups significantly differed in terms of demographics and clinical characteristics. As an example, although we considered only subjects older than 50 years, patients with pachychoroid disease were significantly younger than those with neovascular AMD, which is in agreement with previous reports^16–18^.

As stated above, differentiating neovascular AMD and MNV-associated pachychoroid disease may be challenging as these two disorders may share common features on clinical examination. In addition, as per conventional beliefs, the prevalence of pachychoroid disease complicated by exudative MNV have been historically thought to be low in Caucasian subjects older than 50 years. Based on these assumptions, we additionally investigated the rate of misdiagnosis of pachychoroid disease complicated by exudative MNV as neovascular AMD at our referral retina clinic. Noteworthy, 19 out of 123 patients initially diagnosed as neovascular AMD were successively revealed to be affected by pachychoroid disease. Therefore, approximately 15.4% of eyes deemed to have neovascular AMD based on dilated eye examination and multimodal imaging assessment by retinal specialists or general ophthalmologists had characteristics that indicated pachychoroid disease as revealed by trained raters. Of note, undiagnosed pachychoroid disease with exudative MNV was associated with older patient age, this suggesting that physicians less closely scrutinize the retinal images for signs of pachychoroid in older patients who are more frequently affected by AMD. Our findings suggest that retinal specialist, and more in general ophthalmologists, could benefit from better training in identifying pachychoroid disease.

An appropriate differential diagnosis between neovascular AMD and pachychoroid disease complicated by exudative MNV is important as it may influence disease management. Intravitreal administration of anti-VEGF agents is currently the first-line therapy for neovascular AMD and evidences suggest that this treatment may be also beneficial in eyes with pachychoroid disease complicated by exudative MNV. In the MINERVA study^27^, 22 eyes with MNV secondary to CSC were treated with ranibizumab and experienced a gain of 6.6 letter in visual acuity at the 2-month follow-up visit, in contrast with a gain of 1.6 letter in the sham group. Furthermore, in contrast to neovascular AMD, vPDT is still widely used in combination with intravitreal anti-VEGF treatment in pachychoroid disease cases^16,28^. Assuming that the development of MNV in pachychoroid disease is thought to be related to outer retinal ischemia secondary to choroidal thickening and hyperpermeability, vPDT is aimed at interrupting processes driving the pathogenesis and development of MNV owing to the reduction in choroidal perfusion^16,28^. Therefore, previous studies have proved that the addition of vPDT to anti-VEGF injections may result in an improved control of exudation in eyes with pachychoroid disease complicated by exudative MNV^16,28^.

Avoiding misdiagnosis is also important for a correct determination of patient prognosis. Previous evidences suggest that subjects with pachychoroid disease complicated by exudative MNV have a significantly longer retreatment-free interval than subjects with neovascular AMD after a loading dose of anti-VEGF therapy^7^. The latter finding was speculated to be related to a lower secretion of VEGF in pachychoroid disease, as compared with neovascular AMD^29^. In a post-hoc analysis considering only patients with at least one year of follow-up, we similarly showed that eyes with MNV-associated pachychoroid neovasculopathy are characterized by a lower number of anti-VEGF injections during the first year of treatment, even though this diversity did not reach the statistical significance. In addition, our study did find differences in terms of visual acuity between the 2 groups (pachychoroid disease vs. AMD patients). In details, patients with pachychoroid disease complicated by exudative MNV were characterized by a better visual acuity, at both baseline and 1-year follow-up visits, even after adjusting these comparisons for age. This also directly supports the concept, previously discussed, that patients with MNV-associated pachychoroid disease have a more favorable prognosis.

Our study has limitations that must be considered in the evaluation of our findings. A first limitation was the lack of ethnic diversity represented in our population, as our study cohort only included Caucasian individuals. Future studies with a larger ethnic heterogeneity should be considered to evaluate the influence of race on the relative prevalence of pachychoroid disease complicated by exudative MNV and neovascular AMD in a population older than 50 years. However, this has already been investigated in Asian subjects^7^. Moreover, we evaluated only subjects older than 50 years, which may restrict our findings in pachychoroid disease patients (e.g. mean age and prognosis) to this study population. However, our main purpose was to investigate the relative prevalence of pachychoroid disease complicated by exudative MNV and neovascular AMD in subjects older than 50 years, as well as the rate of misdiagnosis.

In conclusion, this study explored the relative prevalence of neovascular AMD or pachychoroid disease complicated by exudative MNV in patients older than 50 years with newly diagnosed exudative MNV. In our study cohort of Caucasian patients, pachychoroid disease was revealed to be a frequent cause of exudative neovascular maculopathy, as this entity may represent up to one quarter of all cases. Furthermore, our results suggest that underdiagnosis of pachychoroid disease complicated by exudative MNV is not uncommon in tertiary eye care. The reasons underlying misdiagnosis of pachychoroid disease as neovascular AMD remain unclear. Cases of misdiagnosis may be secondary, at least in part, to the conventional belief that prevalence of pachychoroid disease complicated by exudative MNV is quite low in Caucasian subjects older than 50 years. However, proper identification of these patients may have implications for their natural course and management. Finally, our results should also be considered for a proper inclusion in clinical trials.

## Data Availability

The data used to support the findings of this study are available from the corresponding author upon request.
